# The role of γ-secretase in familial hidradenitis suppurativa: Implications for pathogenesis and targeted therapy

**DOI:** 10.1016/j.jdcr.2025.11.010

**Published:** 2025-11-19

**Authors:** Bethel Desta, Ekaterina Korytnikova, Madeleine Tessier-Kay, Tim Klufas, Jun Lu, Akua Sarfo, Albert E. Zhou

**Affiliations:** a University of Texas Medical Branch, John Sealy School of Medicine, Galveston, Texas; b Department of Dermatology, University of Connecticut School of Medicine, Farmington, Connecticut

**Keywords:** familial HS, gamma-secretase, HS, nirogacestat, pathogenesis, subtypes, therapies

*To the Editor:* With the rising number of reports describing the onset of hidradenitis suppurativa (HS) after initiation of γ-secretase inhibitor (GSI) nirogacestat,^1^^,^^2^ these cases highlight an emerging cutaneous adverse of targeted therapy, raising questions about the role of γ-secretase inhibitors in HS pathogenesis.

While its pathogenesis remains incompletely understood, HS is recognized as a multifactorial disease divided into subtypes, including sporadic, syndromic, HS Plus, and familial hidradenitis suppurativa (FHS).^3^ Some genetic mutations serve protective roles, but in FHS, monogenic inheritance accounts for less than 7% of cases and is often due to autosomal dominant mutations in the Notch and γ-secretase pathways, producing a severe, comedo-predominant phenotype.^3^

Mutations in genes encoding components of the γ-secretase complex, including *NCSTN, PSENEN,* and *PSEN1*, are seen in a subset of FHS cases. Disruption of γ-secretase activity impairs Notch signaling, leading to abnormal follicular differentiation and inflammation that underlie HS pathogenesis (Fig 1).^3^ Desmoid tumor pathogenesis, driven by overactivation of the Notch signaling pathway, can be suppressed by GSIs such as nirogacestat that block Notch receptor cleavage.^3^ The ability for nirogacestat to induce follicular lesions in unaffected individuals underscores its role in follicular homeostasis and suggests that drug-induced or ‘acquired’ HS may operate through a similar pathway. In a phase III trial of adults with desmoid tumors, 9% of patients treated with nirogacestat developed HS.^4^ Another study reported that in 12 patients receiving GSIs, 75% of those developed follicular and cystic lesions at typical HS sites with similar clinical and histopathological features.^5^ Many were managed with topical or systemic antibiotics, but 42% underwent local surgical intervention. Discontinuation of GSIs led to resolution.

**Fig 1:**
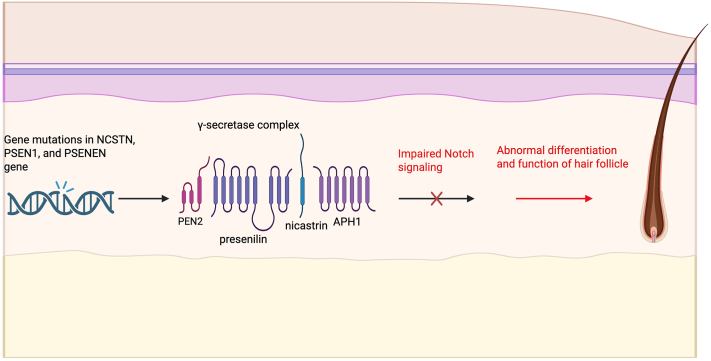
Mutations in the *NCSTN*, *PSEN1*, and *PSENEN* genes, which code for 3 of the 4 essential subunits of the γ-secretase complex (nicastrin, presenilin 1, and PEN-2, respectfully) are linked to familial forms of hidradenitis suppurativa (HS). The γ-secretase complex is a membrane-bound protease responsible for cleaving and activating Notch receptors. Mutations in these subunits impair γ-secretase function, preventing proper Notch receptor activation and leading to disrupted Notch signaling. This disruption results in increased epidermal proliferation and abnormal differentiation of hair follicle keratinocytes and dysregulated local immune responses. Together, these changes contribute to follicular occlusion, chronic inflammation, and the development of nodules and sinus tracts that characterize HS. Created in https://BioRender.com.

These latest reports of acquired HS cases describe the development of HS in patients with no previous history of disease after starting GSI therapy.^1^^,^^2^^,^^6^ Interestingly, the management in these 2 cases encompassed a spectrum. In Frantz et al, due to the clinical trial, the patient was limited to laser hair removal, topical clindamycin, benzoyl peroxide wash, and intralesional steroid injections.^1^ Lesions also presented in a pattern typical of classic HS. In contrast, the patient described by Gutierrez et al developed eruptive milia in addition to HS nodules and was treated with topical therapies and antibiotics but ultimately discontinued the GSI due to worsening symptoms.^2^ In both cases, the cutaneous disease worsened with prolonged GSI use and required surgical deroofing and incisions and drainages. Neither case used biologics, but in a seperate report, secukinumab had to be initiated because of the marked inflammatory features of the lesions.^6^

The temporal relationship, while supportive of a causative role, and whether this subtype should be managed similarly to other forms of HS have yet to be fully elucidated. Given the plethora of GSIs, clinicians should monitor patients on any GSI therapy for dermatologic changes. No therapies to date have been specifically developed for FHS, such as a γ-secretase inducer/modulator despite its genetic basis. Future research, and perhaps additional case reports, may distinguish between inherited versus acquired-HS subtypes, tailored management strategies, and the systemic implications of γ-secretase dysfunction.

## Conflicts of interest

None disclosed.
